# The cold denaturation of IscU highlights structure–function dualism in marginally stable proteins

**DOI:** 10.1038/s42004-018-0015-1

**Published:** 2018-04-05

**Authors:** Robert Yan, Paolo DeLos Rios, Annalisa Pastore, Piero Andrea Temussi

**Affiliations:** 1 Department of Basic and Clinical Neurosciences, King’s College London, London SE5 9RX, UK; 2 Institute of Physics, School of Basic Sciences and Institute of Bioengineering, School of Life Sciences, École Polytechnique Fédérale de Lausanne-EPFL, Lausanne CH-1015, Switzerland; 3 Department of Molecular Medicine, University of Pavia, Pavia 27100, Italy; 4 Dipartimento di Scienze Chimiche, Universita’ di Napoli Federico II, Napoli 80126, Italy

## Abstract

Proteins undergo both cold and heat denaturation, but often cold denaturation cannot be detected because it occurs at temperatures below water freezing. Proteins undergoing detectable cold as well as heat denaturation yield a reliable curve of protein stability. Here we use bacterial IscU, an essential and ancient protein involved in iron cluster biogenesis, to show an important example of unbiased cold denaturation, based on electrostatic frustration caused by a dualism between iron–sulfur cluster binding and the presence of a functionally essential electrostatic gate. We explore the structural determinants and the universals that determine cold denaturation with the aid of a coarse grain model. Our results set a firm point in our understanding of cold denaturation and give us general rules to induce and predict protein cold denaturation. The conflict between ligand binding and stability hints at the importance of the structure–function dualism in protein evolution.

Surprising as it may seem, despite the monumental work by Privalov and associates^1^, the concept of cold denaturation is still not as widely recognized among protein chemists and physicists as it could be expected. It is well established that protein unfolding occurs both at temperatures higher and lower than room temperature. Accurate measurements of protein stability are possible whenever it is possible to measure both unfolding transitions^2,3^, mainly because measurement of the heat capacity difference between the native and denatured states (Δ*C*_p_) is greatly facilitated. However, most proteins undergo the cold denaturation transition at temperatures well below the freezing point of water, making the possibility of accessing this information beyond our reach in most cases. Years ago, we serendipitously discovered a small protein, yeast frataxin (Yfh1), which undergoes unbiased cold denaturation at temperatures above the water freezing point^4^ and used it as a system to test the influence on protein stability of a variety of external factors^2,5–7^.

Is it possible to identify other similar cases? In other words, are there specific properties that make proteins more prone to undergo cold denaturation? Cold denaturation is often attributed to a variety of physical features, which are mainly linked to properties of the medium rather than to intrinsic molecular attributes of the protein under study^8–12^. In particular, the mechanism of cold denaturation proposed by Graziano^11^ highlights the central role of water with respect to other possible solvents. We have recently shown that, in the case of Yfh1, the easy onset of cold denaturation correlates with the presence, on the surface of the protein, of an electrostatic hinge^13^. Four acidic residues are spatially close in Yfh1. Their mutual repulsion creates electrostatic frustration, which favors the entrance of water molecules at low temperature and eventually causes the onset of cold denaturation. An interesting observation is that these residues are semi-conserved in the whole frataxin family since they are essential for the function of these proteins^14^.

Here, we reconsider the previously characterized protein, IscU, which undergoes cold denaturation above zero degrees without the need of denaturing agents^15^. *E. coli* IscU is a small protein whose cellular role is to act as a scaffold for iron–sulfur clusters^16^, essential prosthetic groups common to many proteins involved in redox potential mechanisms^17^. As Yfh1, IscU is negatively charged, marginally stable and undergoes cold denaturation only in the absence of iron–sulfur clusters or zinc and at low ionic strength. We address the question of whether it is possible to understand the structural determinants of cold denaturation by showing that the behavior observed in Yfh1 is not a singularity: we investigate the possible molecular mechanism of the cold denaturation of IscU. By identifying residues that determine electrostatic frustration of IscU, we find that the presence of functionally important residues is again crucial for determining the cold denaturation of this protein. We support our conclusions by the design of suitable mutants. We explore the theoretical implications of our findings by a coarse grain simulation of protein behavior at high and low ionic strength to generalize our conclusions and provide a general theory of the structural requirements that determine cold denaturation. In addition, we find that the relationship between the function of IscU, that concentrates negative charges within a small surface area, and its marginal stability is a further indication of the widespread dualism between structure and function in proteins.

## Results

### IscU has an electrostatic frustration gate

We first looked whether we could identify potential frustrated regions of IscU, which could cause effects like those of the electrostatic hinge in Yfh1. IscU is a scaffold for iron–sulfur clusters, which are transiently mounted on this protein and distributed to other acceptors. To carry iron–sulfur clusters, the protein contains three highly conserved cysteines (Cys37, Cys63, and Cys106) and a histidine (H105) on a tip of the approximately ellipsoidal shape of IscU^18^. When Cys residues are not involved in holding the iron–sulfur cluster or in binding a zinc ion, i.e., in the apo protein, they are at a distance that would result in mutual repulsion at neutral pHs and above. We thus formulated the hypothesis that they could be the key of the cold denaturation of the protein.

### Mutations and protein fold

**Fig. 1:**
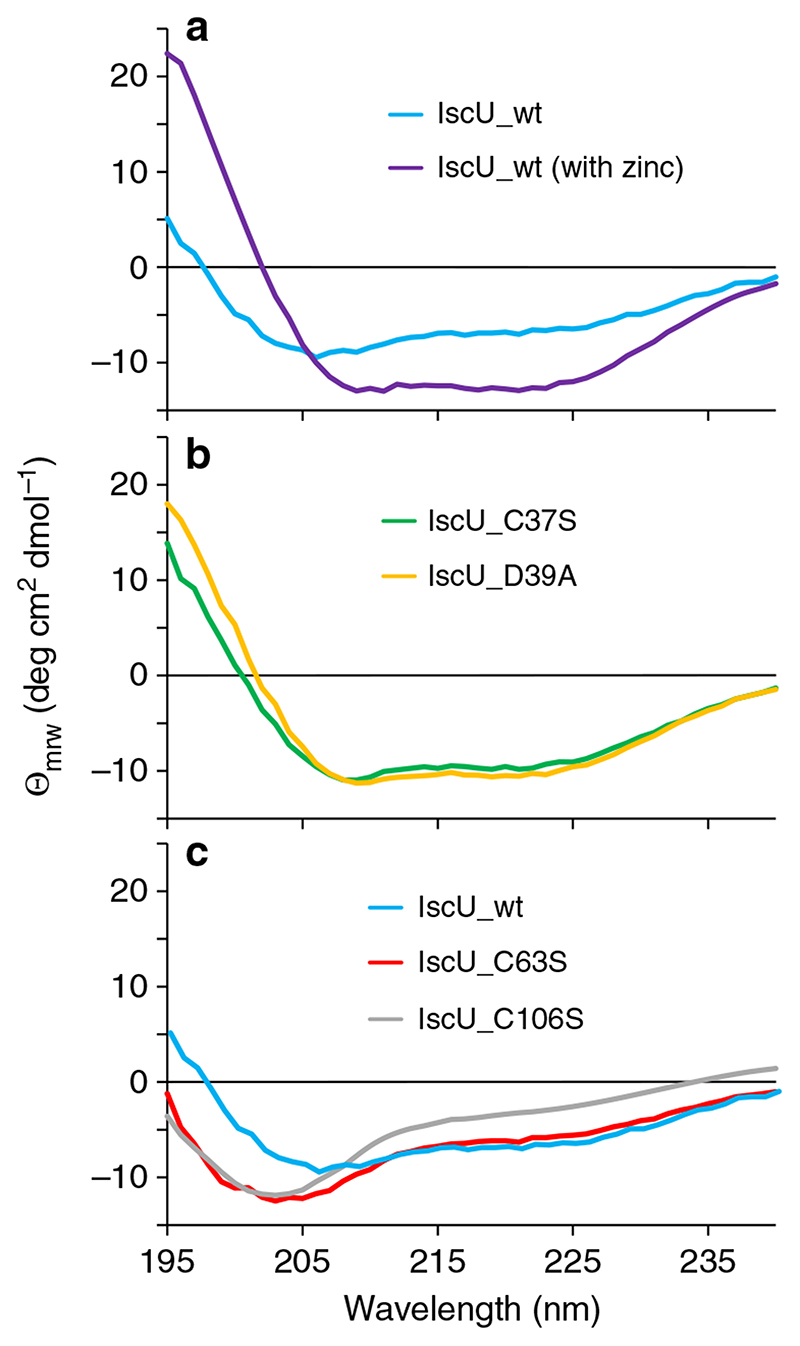
Comparison of the far UV CD spectra of wild-type IscU and those of four mutants. **a** Wild-type IscU with (violet) and without (pale blue) Zn^2+^; **b** IscU_C37S (green) and IscU_D39A (yellow); **c** wild-type IscU without Zinc (pale blue), IscU_C63S (gray), and IscU_C106S (red). Apart from the sample corresponding to one of the wild-type IscU, all samples do not contain zinc. Values on the vertical axis are in multiples of 10^3^

To test our hypothesis, we produced four IscU single mutants, namely IscU_C37S, IscU_C63S, IscU_C106S, and IscU_D39A, and characterized them by far UV circular dichroism (CD) spectroscopy to ensure that they are correctly folded. Comparison of wild-type CD spectra of IscU with and without zinc ion (Fig. 1a) is consistent with the marginal stability of this protein in the apo form, whereas the zinc ion stabilizes the fold.

The far UV CD spectra show that two of the mutations (IscU_C37S and IscU_D39A) lead to an apparent stabilization, as suggested by the similarity of their spectra to that of the wild-type protein containing the zinc ion (Fig. 1a, b). A calculation of secondary structure content (Supplementary Table 1) shows that the architecture of these two mutants is closer to that of IscU_wt in the presence of Zinc. The other two mutations (IscU_C63S and IscU_C106S) destabilize the protein, as indicated by the appearance of their spectra, typical of unfolded proteins (Fig. 1c). To substantiate these qualitative observations, we performed a quantitative study as a function of temperature, corroborated by an original analysis of the data.

### Mutations have a profound influence on the stability of IscU

**Fig. 2:**
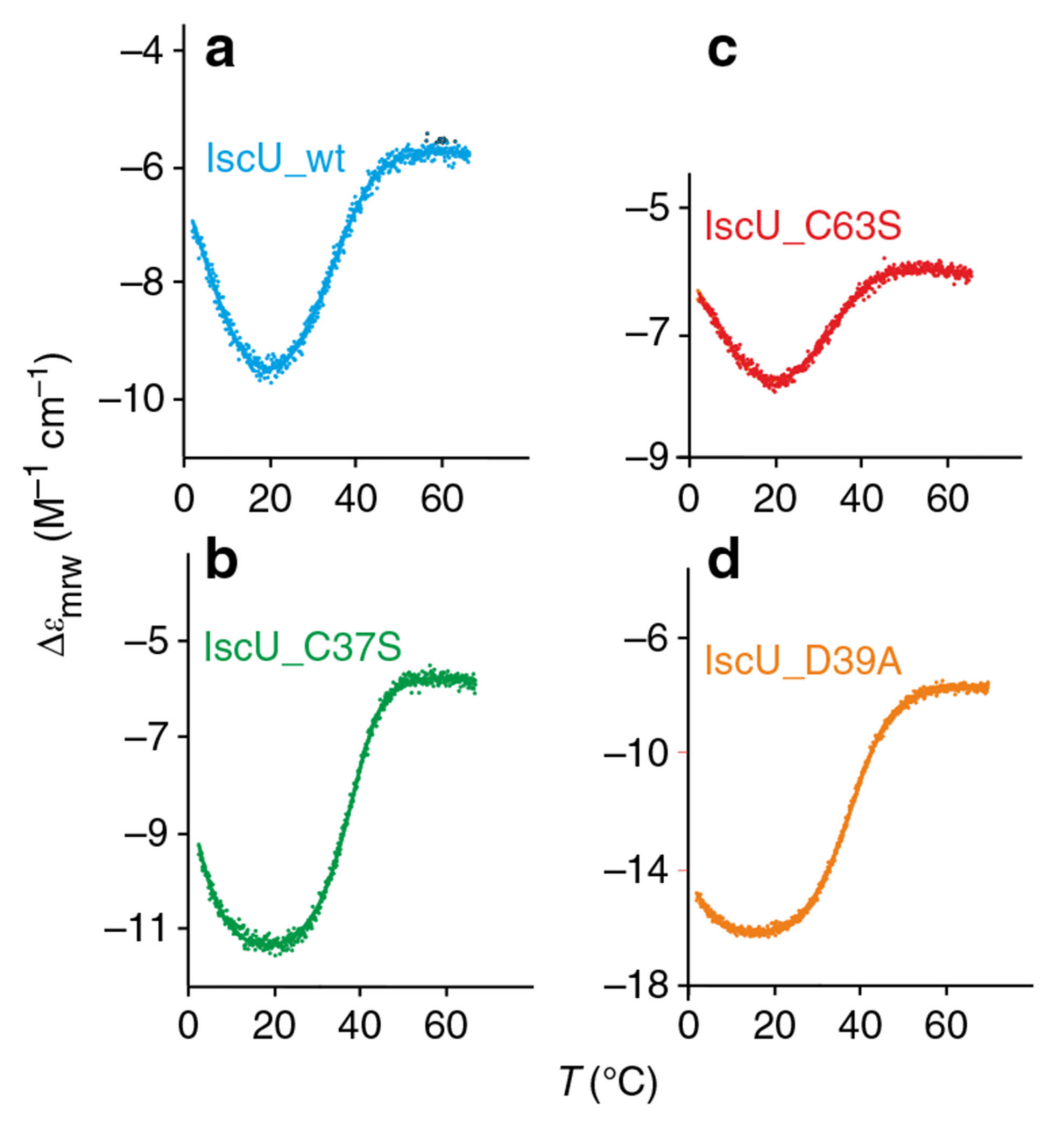
Thermograms of IscU and of its mutants. **a** IscU_wt (no Zn^2+^, pale blue); **b** IscU_C37S (green); **c** IscU_C63S (red); and **d** IscU_D39A (yellow). All thermal denaturation curves were obtained starting from solutions of 90 to 160 μg/ml protein in a 20 mM Tris-HCl buffer at pH 8.05, 0.5 mM TCEP, by monitoring the CD intensity at 222 nm as a function of temperature in the temperature range 2–70 °C. Note that the samples do not contain salt other than the buffer

We measured the thermal denaturation curves of 90–160 μg/ml solutions of each protein to gauge the relative populations of folded and unfolded species of IscU and of its mutants, by recording the CD intensity at 222 nm as a function of temperature in the temperature range 2–70 °C (Fig. 2). From the changes in the thermograms, it is possible to evaluate that both the C37S and the D39A mutations induce a sizeable increase in the population of the folded species. Surprisingly, mutations C63S and C106S lead to constructs that are even less stable than the wild-type protein (Figs. 1 and 2). This finding is not necessarily linked to a change in the potential electrostatic gate, because the partial charges of C63 and C106 are already compensated, in the wild-type protein, by the positive charge of adjacent K103. Rather, it is possible that the smaller sterical encumbrance of the oxygen atom of Ser with respect to the sulfur atom of Cys plays a decisive role.

**Fig. 3:**
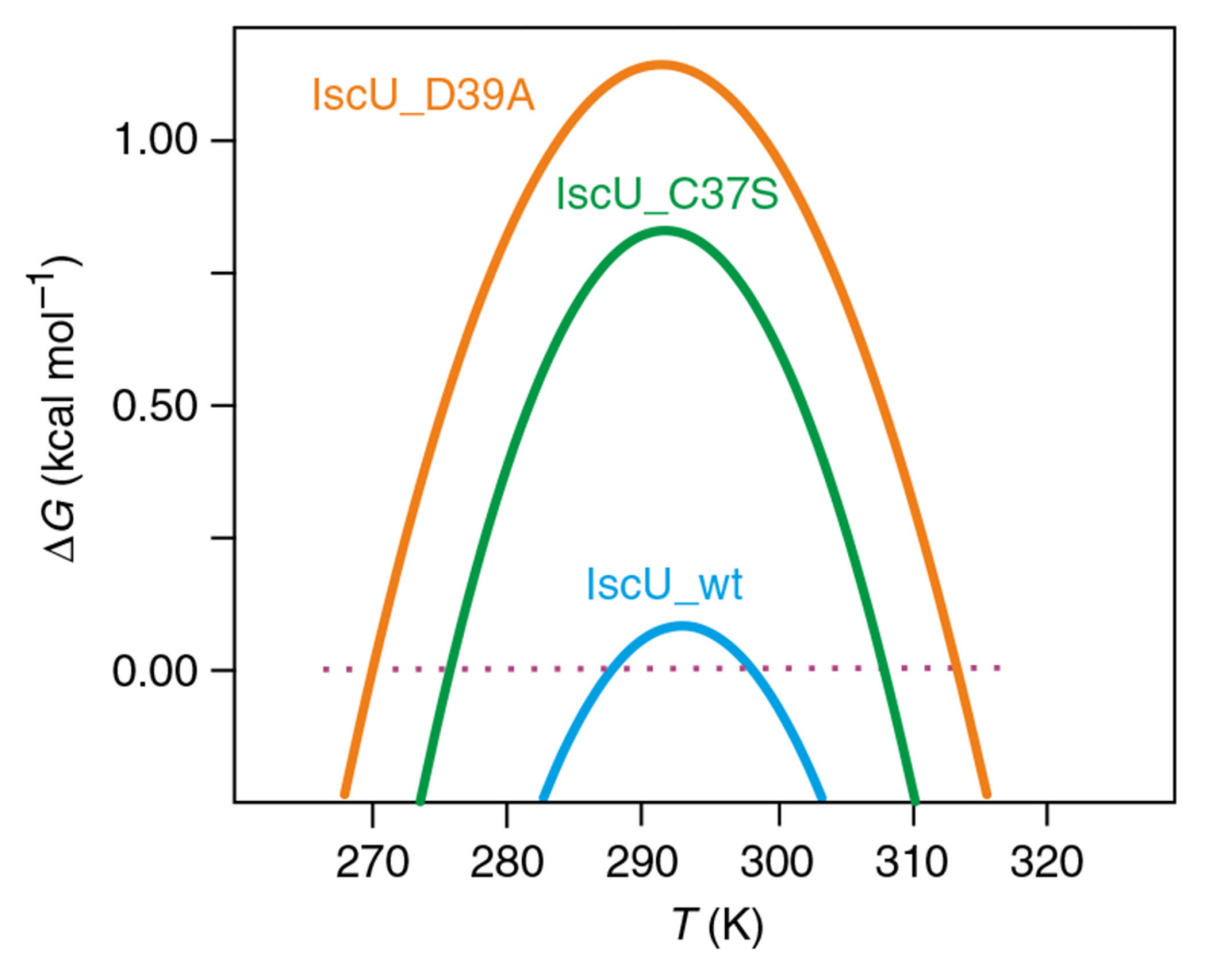
Stability curves of IscU and mutants. Stability curves of IscU correspond to the thermograms of Fig. 2: IscU_wt (pale blue), IscU_C37S (green), and IscU_D39A (yellow). Unfolding temperatures at high (*T*_m_) or low (*T*_c_) temperature are identified by the intersection of the curve with the Δ*G* = 0 line. All attempts to calculate the stability curve of IscU_C63S led to thermodynamic parameters without physical meaning

Fitting of the CD thermal unfolding curves yields stability curves (Fig. 3), from which it is possible to retrieve thermodynamic data (Table 1).

A deeper analysis of the influence on stability of different mutations is appropriate, also because the population of the folded form of wild-type IscU at room temperature is less than 100%^19^. If we assume the existence of a simple two-state equilibrium between folded and unfolded forms, the population of the folded form at any temperature, f_F(T)_, is a function of Δ*G* ^0^ (*T*), the Gibbs free energy for unfolding, as given by Eq. (1) in ref. ^5^.

(1) $$\text{Δ}G=\text{Δ}H_{\text{m}}\;\left(1-\frac{T}{T_{\text{m}}}\right)+\text{Δ}C_{\text{p}}\left\{\left(T-T_{\text{m}}\right)-T\ln\left(\frac{T}{T_{\text{m}}}\right)\right\}.$$

**Fig. 4:**
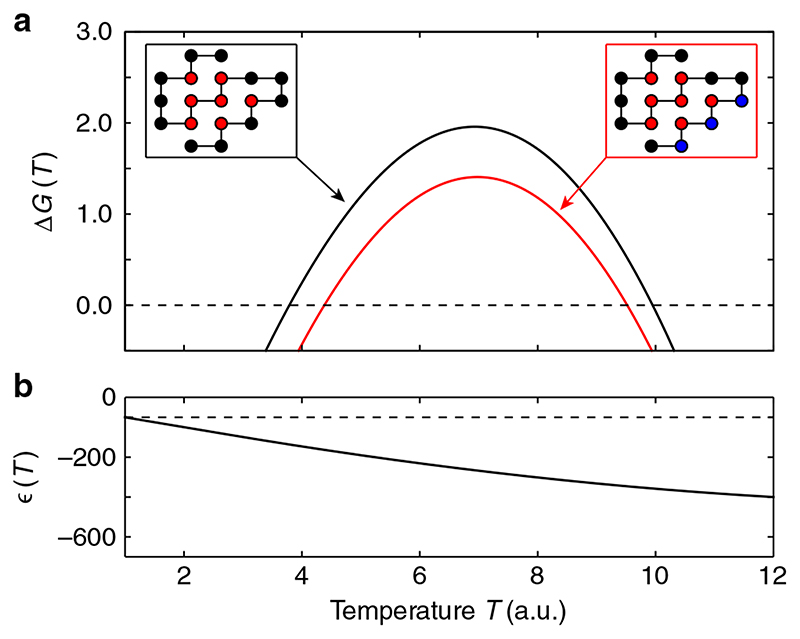
Coarse grain model of cold denaturation. **a** Free energy difference between the native state and the non-native ensemble as a function of the temperature. The black curve corresponds to “high-salt” conditions, when negatively charged residues can be considered just as non-charged polar ones. The red curve corresponds to the “low-salt” conditions, when electrostatic repulsion plays a role. In the left inset, the native state is represented, with hydrophobic residues (H, red) polar, non-charged ones (P, black). In the right inset, the negatively charged residues (C) are highlighted in blue. **b** The HH energy function as a function of the temperature used as described in the “Methods” section

It proved impossible to calculate values for IscU_C63S and IscU_C106S because of the extreme instability of these constructs. For IscU_C106S, it was not even possible to record a thermogram. For IscU_C63S, when attempting to calculate the stability curve from the thermogram of Fig. 4, we obtained thermodynamic parameters with no physical meaning. In addition to the values of Δ*C*_p_, Δ*H*_m_, Δ*S*, *T*_m_, and *T*_c_, Table 1 reports also the values of the area under the stability curve of each construct (I). We have recently proposed^3^ the use of a single parameter to gauge thermal stability: the area of the stability curve between the two unfolding temperatures. To compare areas, we can take the integral of the equation of the stability curve (1) and calculate its value between *T*_c_ and *T*_m_. The integral of Eq. (1) between *T*_c_ and *T*_m_ is given by:

(2) $$\begin{array}{lll} \text{I} & = & \frac{1}{2}\left(\text{Δ}H_{\text{m}}T_{\text{m}}\right)-\frac{1}{4}\text{Δ}C_{\text{p}}\;T_{\text{m}}^{2}+\left(\text{Δ}H_{\text{m}}\frac{T_{\text{c}}^{2}}{T_{\text{m}}}\right)\\ & & -\frac{1}{2}\text{Δ}C_{\text{p}}T_{\text{c}}^{2}\ln\frac{T_{c}}{T_{\text{m}}}+T_{\text{m}}\text{Δ}C_{\text{p}}T_{\text{c}}-\frac{3}{4}\text{Δ}C_{\text{p}}T_{\text{c}}^{2} \end{array}$$

It is easy to grasp that this parameter is an assessment of the global thermodynamic stability within the entire temperature range of protein stability, rather than at a single temperature. This new parameter yields a better estimate of protein stability^3^ particularly in instances when the proportionality between ΔΔ*G* and Δ*T*_m_ does not hold^20^.

### A generalized coarse grain model of cold denaturation

How general is the model of cold denaturation linked to an electrostatic gate? To generalize the protein requirements that allow the observation of cold denaturation above water freezing, we investigated protein behavior from a theoretical point of view. To test the hypothesis that a localized cluster of like charges could reduce the stability of a protein and thus increase its cold denaturation temperature in low-salt conditions, we used a lattice model of proteins. Despite their simplicity, compared to more detailed and realistic all-atom descriptions of proteins, lattice models have been historically helpful to rationalize experimental observations by means of a few controlled ingredients^21^. Coarse grain models have, for instance, been instrumental to clarify the sequence–structure relationship (protein design), highlighting that indeed only an exponentially small set of compact structures can correspond to the compact, native state for many more sequences^22,23^, providing a rationale for the observed small number of protein folds, when compared with the exponentially larger number of protein sequences observed in nature. We thus borrowed a number of crucial ingredients, previously developed for lattice models aimed at reproducing both warm and cold protein denaturation, and extended them to take into account electrostatic interactions in both high and low ionic strengths. The details of the model are provided in the “Methods” section.

We chose an arbitrary polypeptide chain of 18 amino acids, with the sequence PCHCHCPPHHHHPPPHPP (H are hydrophobic amino acids; P are polar uncharged amino acids; C are charged amino acids, all with the same charge), which summarizes essential features shared by the IscU and Yfh1 sequences. The most probable conformation (left/right insets in Fig. 4a) is compact, with all hydrophobic amino acids segregated in the core, as it is typical of proteins. We then computed the free energy difference between the native state and the non-native ensemble as Δ*G* = *k*_B_ *T* ln(*P*_native_/*P*_non-native_) at high (black line) and low (red line) ionic strengths, where P_native_ is the Boltzmann probability of the native state, whereas *P*_non-native_ is the sum of the Boltzmann probabilities of all conformations different from the native one. As it can be seen, the presence of negative charges close to each other in the native state leads to the partial destabilization of the system (although the depicted compact conformation still corresponds to the native state). The free energy difference between the native and non-native ensembles, Δ*G*, decreased in our model in the presence of unscreened clusters of like charges, the cold denaturation temperature increased and the warm denaturation temperature decreased, as observed in experiments (Fig. 3). This modeling approach provides a confirmation that the presence of clusters of like charges on the surface of proteins can reduce the range of stability of the native state of proteins in low-salt conditions, as observed in experiments on frataxin and IscU. As a side observation, that helps understanding the phenomenon of cold denaturation, we point out that the cold denaturation of the sequence analyzed here takes place at a temperature *T*_C_, where the hydrophobic interaction is still attractive (Fig. 4b). In this model, thus, cold denaturation takes place because, as temperature decreases, the attractive nature of the HH interaction weakens faster than the entropic weight of the non-native ensemble.

## Discussion

It is difficult to study cold denaturation of most proteins because it occurs at temperatures lower than the freezing point of water. Observation of cold denaturation generally requires a destabilization of the protein, either by chemical or physical means or by introducing mutations that destabilize the protein fold^24–27^. (Apo)-IscU and Yfh1 are two unusual proteins whose cold denaturation can be observed directly at temperatures above water freezing^4,15^, with the only proviso that ionic strength is kept at the lowest possible value^4,15^. These two proteins constitute ideal tools to investigate potential causes of the origin of cold denaturation on the protein side. What do (apo)-IscU and Yfh1 have in common? The most obvious common feature is that both proteins exist as an equilibrium mixture of folded and unfolded species at room temperature in the absence of salt, with a sizeable population of the unfolded species (around 30%); this circumstance greatly favors the direct observation of the cold melting point because the stability curve of a marginally stable protein has a maximum Δ*G* value (at *T*_S_) rather close to zero (the intersection point for the two unfolding transitions), but this is not sufficient: not all marginally stable proteins have a cold denaturation temperature (*T*_C_) above zero degrees. In this paper, we searched for more specific molecular features that favor the observation of cold denaturation at temperatures above water freezing, focusing on properties of the protein under investigation rather than on more general solvent effects. Specifically, we chose to search for the existence of an electrostatic gate, which could favor the entrance of water molecules in the core of the protein, as found in Yfh1^13^. We found it in four residues evolutionary conserved and responsible for the function of IscU as scaffold of iron–sulfur clusters. They face each other in the structure.

**Fig. 5:**
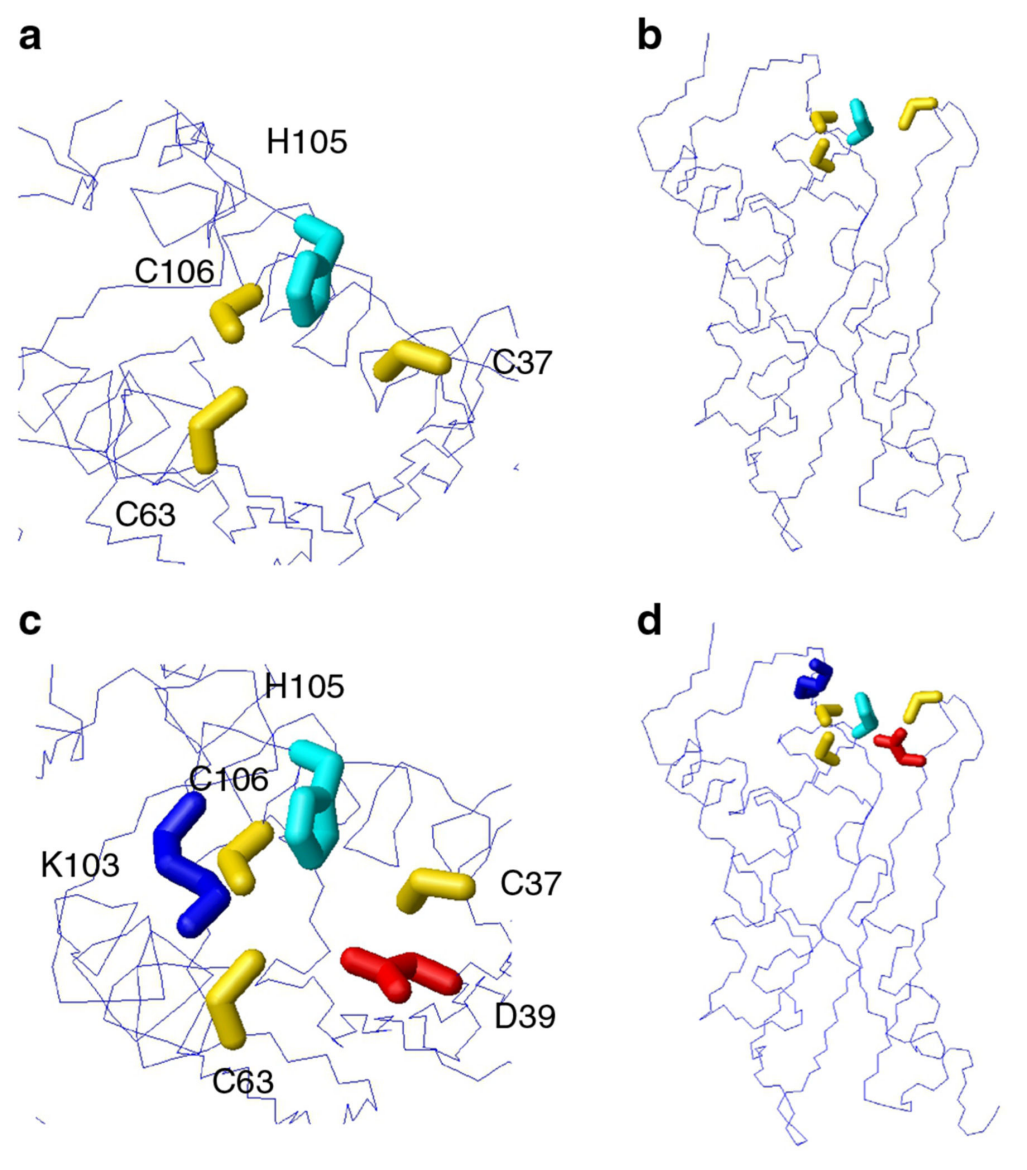
Potential electrostatic gate of IscU. **a** Molecular model of IscU corresponding to the X-ray structure (pdb id 3lvl), showing residues involved in iron–sulfur binding; **b** side view of the model shown in **a**. **c** Same model showing additional charged residue close to the iron–sulfur binding residue; **d** side view of the model shown in **c**. The backbone is represented as line. Key residues are heavy atoms side chains, shown as neon. All models were generated with MOLMOL^36^

We observed that C37S and D39A mutations stabilize the protein considerably, as expected by a decrease in the repulsion among like charges. At variance with the case of Yfh1, the stabilization obtained with these mutants is not markedly asymmetric in favor of cold denaturation, as was the case for Yfh1 mutants. The unique behavior of C63S and C106S came partially as a surprise. We can try to rationalize the results in structural terms. The configuration of the potential electrostatic gate of IscU is clearly more complex than that of Yfh1 (Fig. 5). In addition to the three partially charged cysteines, the protein tip hosts an acidic residue (D39), which can enhance the repulsion among negative charges, a histidine residue (H105) which can partially compensate a small fraction of the negative charge and, most of all, a lysine residue (K103), which is close to two of the cysteines (C63 and C106). The role of D39 has been known for a long time: it is believed that its presence may help to render the binding of an iron–sulfur cluster only transient, as it is necessary in a protein whose function is to deliver the cluster to other target proteins. It has been reported that substitution of D39 with an Ala residue makes binding of the iron–sulfur cluster more stable^28^, hinting at a stabilization of the protein. On the other hand, the presence of K103 close to the cluster-binding residues could divide the four negatively charged residues into two clear classes: the negative charges of C63 and C106, on one side, are checked by K103, whereas C37 and D39 may constitute the better part of an electrostatic hinge. In fact, within a small surface area, there is an excess of negative charge that may favor the entrance of water molecules toward the hydrophobic core and thus enhance unfolding by cold denaturation.

**Fig. 6:**
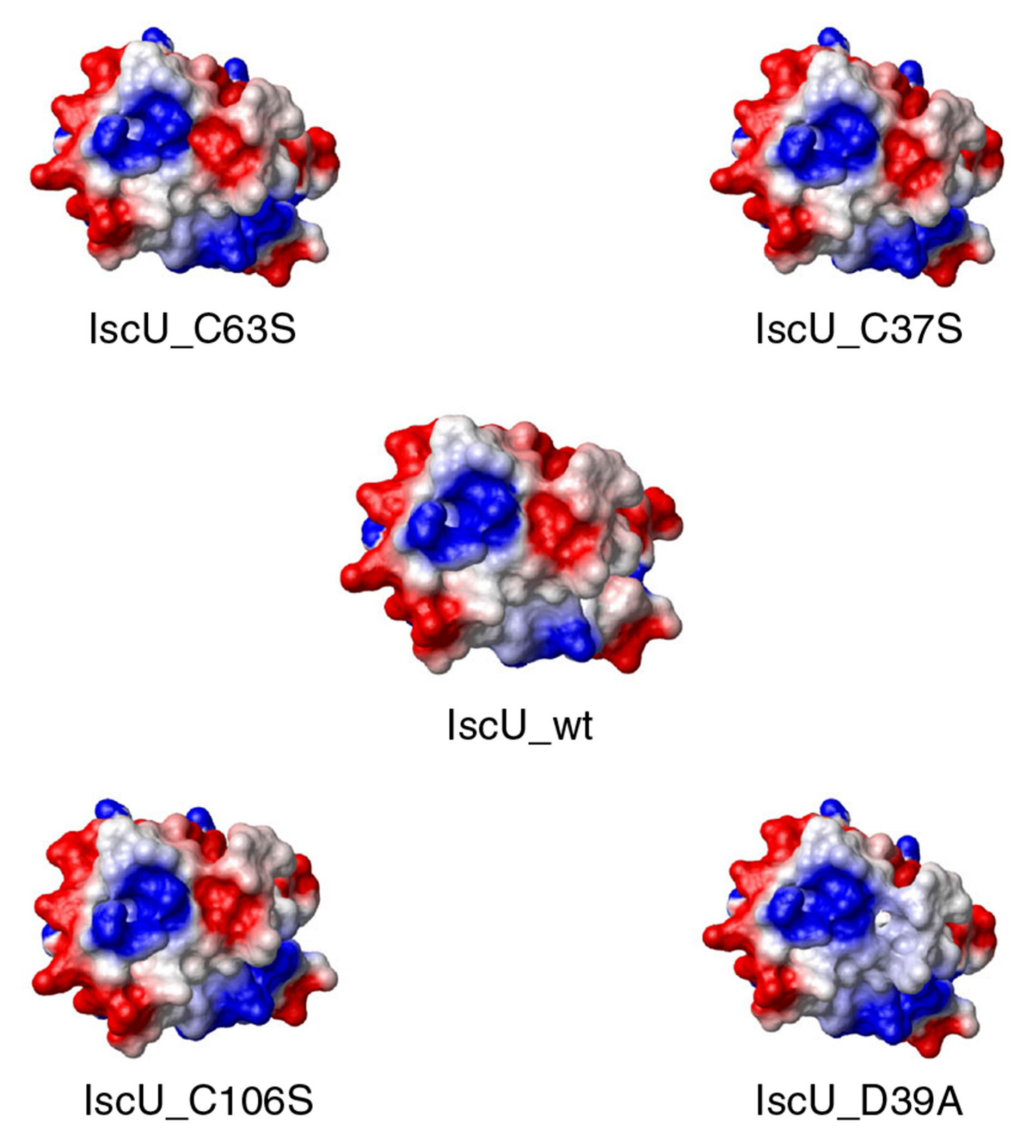
Comparison of the electrostatic surface of IscU and those of four potential mutant constructs. The view is the same as the top views of Fig. 5 (**a**, **c**). The electrostatic surfaces were calculated with MOLMOL^36^. Negative values are shown in red, positive values are shown in blue

Accordingly, if we compare the electrostatic surface of wild-type IscU with those of the mutants (Fig. 6), we notice that only in the case of IscU_D39A, the region of negative charge disappears altogether. It should be noted, however, that in the case of IscU_C37S, the remaining negative region can be attributed entirely to a single residue (D39) and thus cannot possibly be a gate based on charge repulsion. In the remaining two mutants (IscU_C63S and IscU_C106S), the large negative region characterizing the repulsion of C37 and D39 is not reduced. Based on the electrostatic surfaces, it is fair to predict that, of the possible four mutants, two (IscU_D39A and IscU_C37S) might lead to increased stability, whereas the other two (IscU_C63S and IscU_C106S) should not be stabilized, at least as far as the predicted water leak effect is concerned.

It is important to emphasize that we do not claim to unveil the role of electrostatic repulsion in cold denaturation. Prof. Privalov in an investigation described in detail in ref. ^1^, showed, by means of different physico-chemical probes, that met-myoglobin undergoes cold denaturation, well above 0 °C, around pH 3.8, but not at higher pH values. These results can readily be interpreted as a demonstration of the main role played by electrostatic repulsion. However, these results could not benefit, in the 1990s, of the molecular biology tools. In our investigation, we were able to show that both in Yfh1 and in IscU, the residues that determine cold denaturation are functionally important: the electrostatic hinge in Yfh1 is essential for recognition of the protein partner IscS, while IscU is a scaffold for iron–sulfur clusters and uses the residues we have mutated to bind to the clusters and transfer them to other proteins. This suggests an important dualism between structure and function: in proteins, functional aspects may coincide with features which cause destabilization, aggregation, or even malfunction as previously noticed in another contest^29^. This important lesson may be valuable in the future to inform identification of other proteins, which undergo detectable cold denaturation.

Despite the partial decrease of repulsion among negative charges, mutations C63S and C106S lead to a further marked decrease of the stability of IscU. In both cases, the decreased stability may be attributed to causes different from electrostatic repulsion. Contrary to C37 and D39, in the wild-type protein the partial charges of C63 and C106 do not contribute significantly to the electrostatic gate, because they are compensated by the positive charge of adjacent K103. Rather, it is possible that the smaller sterical encumbrance of the oxygen atom of Ser with respect to the atom of Cys plays a decisive role. In addition, as shown in Fig. 5, the *ε*2N atom of H105, although approximately equidistant from each of the three atoms, may interact specifically with that of the Cys residues (C63) since the CH2–S bond points at the *ε*2N of H105, as illustrated by the X-ray (3LVL) and NMR structures (1R9P).

While the original motivation for the present research was mainly centered on unraveling the molecular determinants of cold denaturation, the results shed light on an even more general and important aspect of proteins. The relationship between the function of IscU, that is holding transiently an iron cluster with the consequent need to concentrate negative charges within a small surface area, may be considered as a further indication of the widespread dualism between structure and function in proteins. In the recent past, we have drawn attention on the possible competition between normal function, misfolding, and aggregation in many proteins involved in neurodegenerative diseases. We argued that it is possible to invoke the dual role played by protein–protein interactions to explain how proteins involved in misfolding diseases can resist aggregation in the cell. A wealth of experimental evidence suggests that normal and aberrant interactions may be viewed as the opposing faces of the same coin. In this context, function represents the competing pathway of aggregation rather than folding.

Many other groups are now highlighting the relevance of structure–function dualism. Prominent is the contribution of Franzese and coworkers. Recent results from this group show, in a different context, that general features of protein structure are related to the optimization of function^30^. In other words, surface conservation found in naturally evolved proteins may be regarded as a way to maximize the number of folding sequences at ambient conditions. In these authors’ most recent paper^30^, they elucidate the role played by water in the selection process of protein sequences. Putting the hydrophobic effect in an evolutionary perspective, they show that many features observed in natural proteins arise naturally if the selection process takes into account the thermodynamic properties of the solvent.

## Methods

### Protein production

Cells overexpressing IscU and its mutants were grown in Luria broth enriched with 8.3 μM ZnSO_4_ as described previously^31,32^. *Bacterial* IscU and the constructs containing each of the four mutations (C37S, C63S, C106S, and D39A) were produced as fusion proteins with a His-tagged GST and purified by affinity chromatography using Ni-NTA agarose gel (QIAGEN), as previously described^32,33^. Purification was carried out in the presence of 0.5 mM TCEP. Constructs were cleaved overnight from GST by TEV protease and further purified by a Ni-NTA agarose column and gel-filtration chromatography on a Superdex 75 16/600 pg column (GE Healthcare). Protein purity was checked by SDS-PAGE. To remove zinc, proteins were treated with a 500-fold excess of EDTA followed by desalting with PD-10 columns (GE Healthcare) equilibrated with CD sample buffer (20 mM Tris pH 8.05, 0.5 mM TCEP) to separate protein from EDTA and zinc, as described previously^15^.

### CD

CD measurements were made for IscU and each of the its mutants at concentrations of 90–160 μg/ml in 20 mM TRIS buffer at pH 8.05, 0.5 mM TCEP in 1 mm path length cells (Hellma). Wavelength scans were recorded between 260 and 190 nm at 25 °C. Thermal unfolding curves were obtained by monitoring the ellipticity at 222 nm using a Jasco J-815 CD spectro polarimeter equipped with a Jasco CDF-4265/15 Peltier unit, with a heating rate of 2 °C/min in the temperature range 2–70 °C.

### Homology modeling

Sequence alignments were generated with ClustalX^34^. Mutant models of C37S-Iscu, C63S-IscU, C106S-IscU, and D39A-IscU were built using the automatic mode of SWISS MODEL^35^ and the IscU structure of the *E. coli* IcsS–IscU complex as template (PDB entry 3lvl).

### IscU mutagenesis

Constructs for the recombinant expression of IscU mutants C37S, C63S, and C106S were generated by mutating the wild-type construct used in previous studies^32^ using Quickchange© (Agilent). Mutagenesis was confirmed by sequencing. The construct for IscU D39A was available from previous studies^15^.

### Coarse grain modeling

We modeled proteins on a two-dimensional grid, where each conformation is a self-avoiding path and where amino acids occupy lattice sites. Each amino acid can be of one of three types: hydrophobic (H), non-charged polar (P), or charged (C) (in keeping with the clusters of negative charges for frataxin and IscU, in the model we only considered like charges, e.g., C = negative). Each conformation is characterized by intrinsic energy and entropy. The intrinsic energy is the sum of contact energies between residues located on nearest-neighbor lattice sites, but non-consecutive along the chain. The functional form of the hydrophobic interaction, shown in Fig.4b, is heuristically inspired by the effective interaction between hydrophobic amino acids that can be obtained from a model of lattice polymers with simplified semi-explicit water, by tracing out the solvent degrees of freedom (ref. ^10^).

The contact energy *ε*_XY_ depends on the pair of chemical species *X* and *Y* (*X* and *Y* = H, P, or C) that occupy the two sites. In particular, *ε*_HH_ = *ε*(*T*), where *ε*(*T*) (depicted in Fig. 4b) is an effective attractive energy that captures the salient features of the hydrophobic effect, namely that it becomes weaker as temperature decreases, and that saturates at higher temperatures, as observed in experiments^1^ and reproduced by simplified models^10,12^. When only one of the two moieties is hydrophobic, the energy gain associated to the hydrophobic effect is reduced: *ε*_HP_ = *ε*_HC_ = *ε*_PP_ = 0.1*ε*(*T*). High-salt conditions (high ionic strength) can be reproduced by assuming that the net charge of C-residues is mostly screened, so that they are similar to polar, non-charged amino acids, and *ε*_CC_ = *ε*_PP._ In low-salt conditions (low ionic strength), instead, the repulsive energy between like charges is *ε*_CC_ = 4. Each exposed amino acid contributes an intrinsic entropy, which is lost upon burial in the protein, corresponding to ln(*q*) (here *q* = 2.5), to account for the freedom of movement of side chains. The chosen sequence, PCHCHCPPHHHHPPPHPP, has been chosen because it satisfies the minimal property associated to protein-like behavior, namely that in a temperature range a single conformation has a statistical weight larger than the cumulated weight of all other conformations. This conformation is associated to the native state. We have then computed the energy and entropy, and the corresponding probability, associated to each of the 5′808′337 conformations accessible to 18-monomers self-avoiding walks on a two-dimensional lattice (excluding global symmetries) in a broad temperature range and in both low- and high-salt conditions. The free energy difference between the native state and the non-native ensembles is Δ*G* = *k*_B_ *T* ln(*P*_N_/*P*_nN_).

## Supplementary Material

Supplementary Information **Supplementary information** accompanies this paper at https://doi.org/10.1038/s42004-018-0015-1.

## Figures and Tables

**Table 1:** Thermodynamic parameters

	IscU_wt	IscU_C37S	IscU_D39A
Δ*C*_p_ (kcal mol^−1^ K^−1^)	1.71	2.01	1.31
Δ*H*_m_ (kcal mol^−1^)	10.5	35.7	30.6
Δ*S* (kcal mol^−1^ K^−1^)	0.0351	0.116	0.0980
*T*_m_ (K)	299	309	315
*T*_c_ (K)	287	275	270
*I* (kcal mol ^−1^ K)	0.86	23	33
*I*/*I*_o_^a^	1	27	38

a *I*_o_ represents the value of *I* for the wild-type protein

## Data Availability

The data that support the findings of this study are available from the corresponding author on reasonable request.
