# Supplementary material for: The cold denaturation of IscU highlights structure–function dualism in marginally stable proteins
Source: Commun Chem. Author manuscript; Available in PMC 2022 Mar 2. (PMC7612454; doi:10.1038/s42004-018-0015-1)
Supplement: Supplementary Information — accompanies this paper at https://doi.org/10.1038/s42004-018-0015-1. [file EMS142169-supplement-Supplementary_Information.pdf]

Suplementary Table 1. Secondary structure estimates[1]

	Wt_IscU_apo	Wt_IscU_apo + Zn	C37S	D39A	method
	%	%	%	%	
**alpha**	16.6	39.8	30.8	34.3	Selcon3
	20.5	36.6	35.3	33.4	Contin-LL
	17	31	36	33.4	cdsstr
**beta**	29.9	14.3	18.7	16.1	Selcon3
	23.7	15.8	16.6	17.5	Contin-LL
	26	29	16	17.5	cdsstr

Supplementary References

1. Whitmore, L. & Wallace, B. A. Protein secondary structure analyses from circular dichroism spectroscopy: methods and reference databases. *Biopolymers* **89**, 392-400 (2008).
